# Comparison Study of Wide Bandgap Polymer (PBDB-T) and Narrow Bandgap Polymer (PBDTTT-EFT) as Donor for Perylene Diimide Based Polymer Solar Cells

**DOI:** 10.3389/fchem.2018.00613

**Published:** 2018-12-10

**Authors:** Tengling Ye, Shan Jin, Cong Kang, Changhao Tian, Xin Zhang, Chuanlang Zhan, Shirong Lu, Zhipeng Kan

**Affiliations:** ^1^MIIT Key Laboratory of Critical Materials Technology for New Energy Conversion and Storage, School of Chemistry and Chemical Engineering, Harbin Institute of Technology, Harbin, China; ^2^Organic Semiconductor Research Center, Chongqing Institute of Green and Intelligent Technology, Chinese Academy of Sciences, Chongqing, China; ^3^Beijing National Laboratory for Molecular Sciences, CAS Key Laboratory of Photochemistry, Institute of Chemistry, Chinese Academy of Sciences, Beijing, China

**Keywords:** polymer solar cells, perylene diimide, charge transport, charge recombination, non-fullerene, wide bandgap, narrow bandgap

## Abstract

Perylene diimide (PDI) derivatives as a kind of promising non-fullerene-based acceptor (NFA) have got rapid development. However, most of the relevant developmental work has focused on synthesizing novel PDI-based structures, and few paid attentions to the selection of the polymer donor in PDI-based solar cells. Wide bandgap polymer (PBDB-T) and narrow bandgap polymer (PBDTTT-EFT) are known as the most efficient polymer donors in polymer solar cells (PSCs). While PBDB-T is in favor with non-fullerene acceptors achieving power conversion efficiency (PCE) more than 12%, PBDTTT-EFT is one of the best electron donors with fullerene acceptors with PCE up to 10%. Despite the different absorption profiles, the working principle of these benchmark polymer donors with a same electron acceptor, specially PDI-based acceptors, was rarely compared. To this end, we used PBDB-T and PBDTTT-EFT as the electron donors, and 1,1′-bis(2-methoxyethoxyl)-7,7′-(2,5-thienyl) bis-PDI (Bis-PDI-T-EG) as the electron acceptor to fabricate PSCs, and systematically compared their differences in device performance, carrier mobility, recombination mechanism, and film morphology.

## Introduction

**Figure 1:**
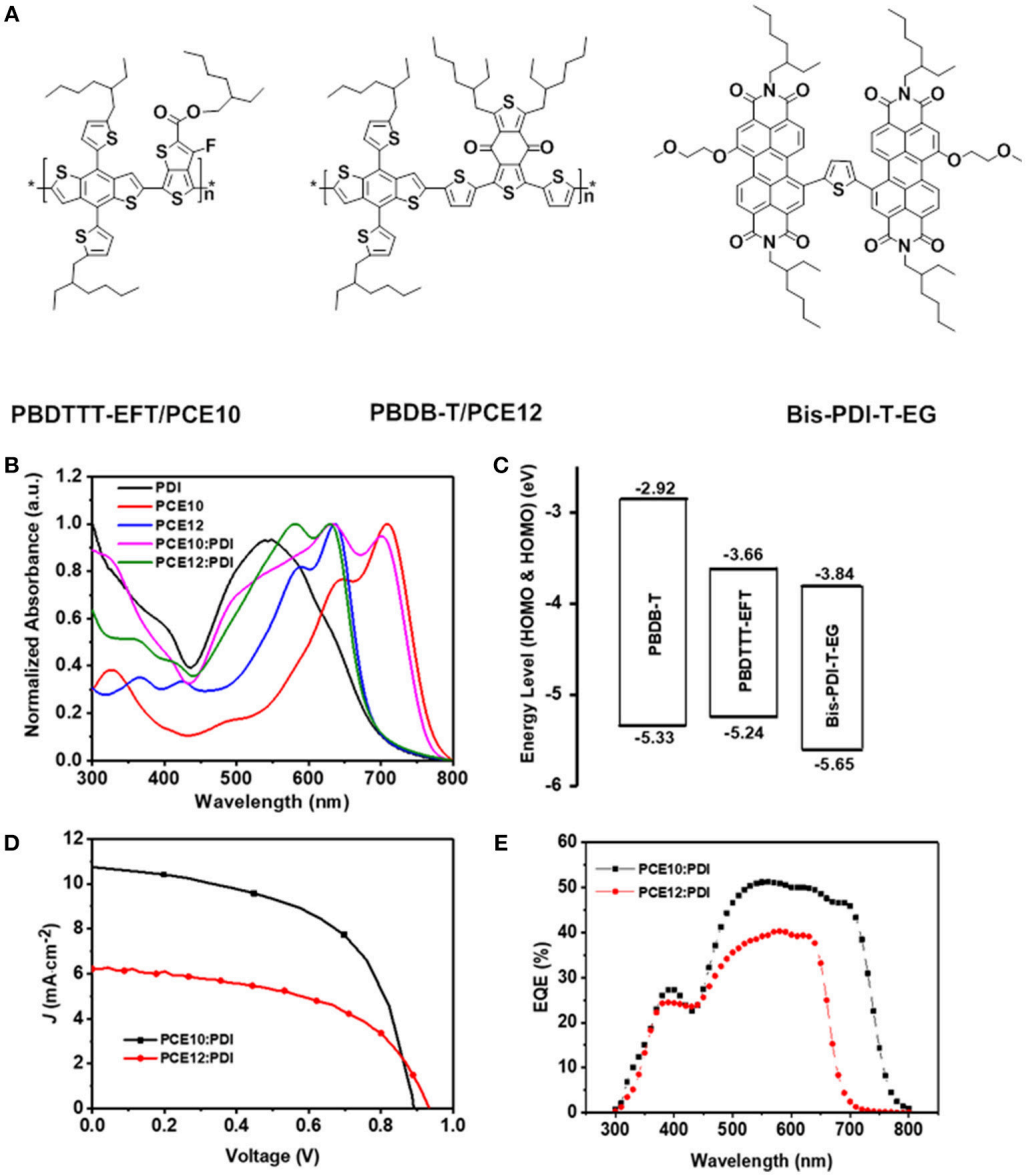
**(A)** The chemical structures of the PBDTTT-EFT/PCE10, PBDB-T/PCE12 and Bis-PDI-T-EG, **(B)** Normalized UV-Vis absorption spectra, **(C)** Energy-level diagrams, **(D)** J–V characteristics and **(E)** EQE spectra of the PSCs based on PCE10/PDI and PCE12/PDI.

Polymer solar cells (PSCs) have been attracting more and more attention in both research and industrial applications due to their unique properties such as solution processability, light weight, low cost, and high mechanical flexibility. High-efficiency PSCs typically employ the bulk heterojunction structures composed of a p-type polymer material and an n-type small molecular material. The most studied combination was a polymer semiconductor donor and a fullerene-based small molecule acceptor, reaching a power conversion efficiency (PCE) of 11.7% (Zhao et al., 2016a). Due to the drawbacks of fullerene-based materials, such as weak absorption in visible and near infrared region, limited tunability of energy levels, and poor morphology stability, further improvement of the PCE in such traditional donor/acceptor combinations is hindered (Eftaiha et al., 2014; Cheng and Zhan, 2016). To overcome these limiting factors, efficient non-fullerene-based acceptors (NFA) have been developed for decades. Yuze Lin (Lin et al., 2015a,b) and co-authors reported a series of NFAs based on highly electron-deficient (3-ethylhexyl-4-oxothiazolidine-2-yl) dimalononitrile (RCN) groups, such as ITIC IEIC and SFBRCN, getting PCE comparable to their fullerene counterparts and opening a new era of PSCs. After that, the first single cell with PCE more than 11% based on polymer/NFA was reported by Wenchao Zhao etc. (Zhao et al., 2016b). The RCN-based NFAs has been also introduced into ternary PSCs and tandem PSCs. The best PCE of single PSCs based on these acceptors has already exceeded 14% up to date (Xiao et al., 2017a; Li et al., 2018) and the optimal efficiency of tandem organic solar cells is up to 17.3% (Cui et al., 2018; Meng et al., 2018). The perylene diimide (PDI)-based small molecules as another kind of promising NFAs have been intensively studied. However, the development of PDI-based NFAs in efficiency is lagging. The main factor preventing PDI-based NFAs to get higher efficiency is that PDI moleculars have the intrinsic tendency to aggregate in solid thin film, where excimers are formed and the process of exciton diffusion/separation is severely limited (Ye et al., 2013). To overcome the aggregation, the design of twist PDI dimer derivatives linked at the imide positions or bay positions were designed. Bis-PDI-T-EG is an example of PDI dimmer linked by a thiophene group at bay position with a bandgap of 1.81 eV. A PCE of 6.1% was achieved when it was blended with PBDTTT-C-T by finely tuning the active layer morphology (Zhang et al., 2013, 2015). Recently, various linkers in PDI–π-linker–PDI type systems were reported, giving PCEs up to 9.5% (Liu et al., 2016; McAfee et al., 2017; Welsh et al., 2018a,b). In addition, the introduction of annulation to PDI molecular is another strategy to significantly improve performance giving PCEs in the range of 7–8% (Sun et al., 2015; Hendsbee et al., 2016; Meng et al., 2016; Dayneko et al., 2018). Although a great amount of work has been done in developing new PDI derivatives to restrict their intrinsic aggregation tendency, little work paid attention to the effect of the polymer donor selection on PDI-based on PSCs. PBDB-T, also named as PCE12, worked modest with PCBM, but performed amazingly high PCE with NFAs (Zhao et al., 2016b). PBDTTT-EFT, also named as PCE10 was found to be efficient with both fullerene-based acceptors and NFAs (Chen et al., 2015; Zhang et al., 2017). Despite the different absorption profiles, the working principle of these benchmark polymer donors with one PDI acceptor was rarely compared. To this end, we compared the PSCs made with PCE12 and PCE10 as the electron donors, and 1,1′-bis(2-methoxyethoxyl)-7,7′-(2,5-thienyl) bis-PDI (Bis-PDI-T-EG) as the electron acceptor. If not otherwise mentioned, we will refer PCE10 to PBDTTT-EFT, PCE12 to PBDB-T, and PDI to Bis-PDI-T-EG. The chemical structures of the PCE10, PCE12 and Bis-PDI-T-EG were shown in Figure 1A. Herein, we compared their differences in device performance, charge carrier mobility, recombination mechanism, and film morphology. The PSCs of PCE12/PDI and PCE10/PDI can give a PCE of 3 and 5.3%, respectively, both with FF about ~50–60%. The hole mobilities of both devices were similar, 3.4 × 10^−4^ and 6.4 × 10^−4^ cm^2^/V s for PCE12/PDI and PCE10/PDI, respectively. However, the electron mobilities were 2.3 × 10^−6^ and 1.2 × 10^−5^ cm^2^/V s, which were much lower than the hole mobilities. The low and unbalanced charge carrier mobilities should be one of the limiting factors for the low FF of these PSCs. By combining photoluminescence (PL) quenching efficiency, light intensity dependent J-V measurements, transient photocurrent and transient photovoltage, we systematically studied the recombination profiles in two systems. Both systems showed a similar extent of bimolecular recombination, while the PCE10/PDI device suffered a severe trap-assisted recombination. The high electron mobility should be the key factor for efficient charge extraction and thus the high performance in PCE10/PDI devices. The mobility was resulted from the morphological difference, i.e., the distinct aggregation and phase separation in the blends. The results indicate that it is important to examine the donor and acceptor aggregation nature when we make choice of donors for Bis-PDI-T-EG based PSCs.

## Results and Discussion

### Optical Properties and Photovoltaic Device Performance

Figure 1B shows the normalized UV-Vis absorption spectra of PCE10, PCE12, Bis-PDI-T-EG and their blend films. In the spectra, there are two distinguished features: (1) the absorption spectra of PCE12 and PDI substantially overlap with each other in the visible range from 450 to 700 nm; (2) the absorption spectra of PCE10 and PDI are well complementary to each other, covering the wavelength range from 300 to 800 nm. Thus, the PCE10/PDI blend film has the potential to harvest more photon energy compared with that of PCE12/PDI, which may result in higher short circuit current (J_SC_) in the devices (Xiao et al., 2017b).

The lowest unoccupied molecular orbital (LUMO) and the highest occupied molecular orbital (HOMO) offsets between the donor and the acceptor are shown in Figure 1C (Zhang et al., 2014, 2015; Zhao et al., 2016b). It is noticeable that the existing energy offsets for electron transfer from donor to acceptor and the hole transfer from acceptor to donor are sufficient in both PCE10/PDI and PCE12/PDI systems. We also notice the HOMO energy difference in PCE10 and PCE12, i.e., the HOMO of PCE12 is about 0.1 eV deeper compared with that of PCE10, which may lead to a higher device open circuit voltage (V_OC_). Proper device performance is expected for both polymer/PDI blends.

We fabricated solar cells in inverted device architecture to check the photovoltaic properties of the two blends. The device performance of optimized PSCs are listed in Table 1, and the optimal current density-voltage (J–V) curves and the corresponding external quantum efficiency (EQE) spectra are displayed in Figures 1D,E. As shown in Table 1, the device made with PCE12/PDI can yield PCEs of 3% in average, with a modest JSC and FF value of 6.15 mA cm^−2^ and 52%, respectively. As predicted from the energy alignment, the VOC is quite high with a value of 0.94 V. On the other hand, the device made with PCE10/PDI can yield markedly better PCEs of 5.3% in average, with a JSC value of 10.61 mA cm^−2^, a FF value of 56% and a VOC value of 0.89 V. The apparent difference in the value of JSC is also observed in the EQE spectra plotted in Figure 1E. The EQE spectra of devices made with PCE10/PDI cover the range from 300 to 800 nm, while the PCE12/PDI devices only cover the spectra range from 300 to 750 nm. Furthermore, the maximum EQE in PCE10/PDI devices is about 10% higher than that of the PCE12/PDI devices. One should note that the integrated J_SC_ from the EQE spectra was within 5% deviation compared to the one measured under solar simulator.

**Table 1:** Device performance of PSCs based on different donors.

**Active layer**	**J_**SC**_(mA cm^**−2**^)**	**V_**OC**_(V)**	**FF**	**PCE (%)**
PCE12/PDI	6.15	0.94	0.52	3.00
PCE10/PDI	10.61	0.89	0.56	5.29

*Values were averaged from 10 working devices*.

To further understand the differences in J_SC_, we performed optical simulation on the maximum J_SC_ in the two blends by considering the internal quantum efficiency (IQE) to be 100% and only relating with absorption. As shown in Figures S1A,B (Margulis et al., 2013), we found that the values of J_SC, max_ of PCE12/PDI and PCE10/PDI were 12.80 and 15.70 mA cm^−2^ at the optimized film thickness and the difference of the calculated Jsc (12.8/15.7 = 0.815) was much closer compared with the measured values (6.15/10.61 = 0.579). The averaged IQE can be estimated as a ratio of measured J_SC_ to maximum theoretical J_SC, max_ obtained as mentioned above by optical simulation. In addition, the IQE can be separated into two contributions which are charge generation efficiency (η_gen_) and charge collection (and transport) efficiency (η_coll_) (Benten et al., 2016):

(1) $$\begin{array}{l} \text{IQE}=\frac{{\text{J}}_{\text{sc}}}{{\text{J}}_{\text{sc},\text{max}}}=\frac{{\;\eta }_{\text{gen}}}{{\;\eta }_{\text{coll}}} \end{array}$$

The average IQE with value of 48% (PCE12/PDI) and 68% (PCE10/PDI) were obtained. We attribute the larger current density deviation of measured J_SC_ and the non-unity IQE to not only the different absorption but also great related with recombination, and charge transport.

### Charge Transport and Recombination

Before examining the charge recombination happened in the devices, we first checked the charge transport by space charge limited current (SCLC) model (Blakesley et al., 2014). The measured dark current density was fitted with the following equation and the parameters are listed in Table 2:

(2) $$\begin{array}{l} J\left(V\right)=\frac{9}{8}\varepsilon_{0}\varepsilon_{r}\mu_{0}\text{exp}\left(0.89\beta \sqrt{\frac{V}{L}}\right)\frac{{\left(V\right)}^{2}}{L^{3}} \end{array}$$

The dark current density and fitting curve are shown in Figures S2A,B. The hole mobilities of 3.4 × 10^−4^ and 6.4 × 10^−4^ cm^2^/V s were obtained for the blends of PCE12/PDI and PCE10/PDI. The electron mobilities of PCE12/PDI and PCE10/PDI were 2.3 × 10^−6^ and 1.2 × 10^−5^ cm^2^/V s, respectively, which were much lower than the hole mobilities. It is worth noting that the imbalance of hole and electron mobilities is likely the origin of significant space charge build-up in the optimized polymer/PDI solar cells, which in turn limits the photovoltaic performance.

**Table 2:** The parameters in the equation of the measured dark current density.

**Definition**	**Variable**	**Units**
Zero-field mobility	μ_0_	cm^2^ V^−1^ s^−1^
Film thickness	*L*	cm
Dark current density	*J*	mA cm^−2^
Voltage	*V*	V
Vacuum permittivity	ε_0_ (88.54 × 10^−12^)	mA s V^−1^ cm^−1^
Dielectric constant	ε_r_ (3)	
Field activation factor	β	cm^1/2^ V^−1/2^

**Figure 2:**
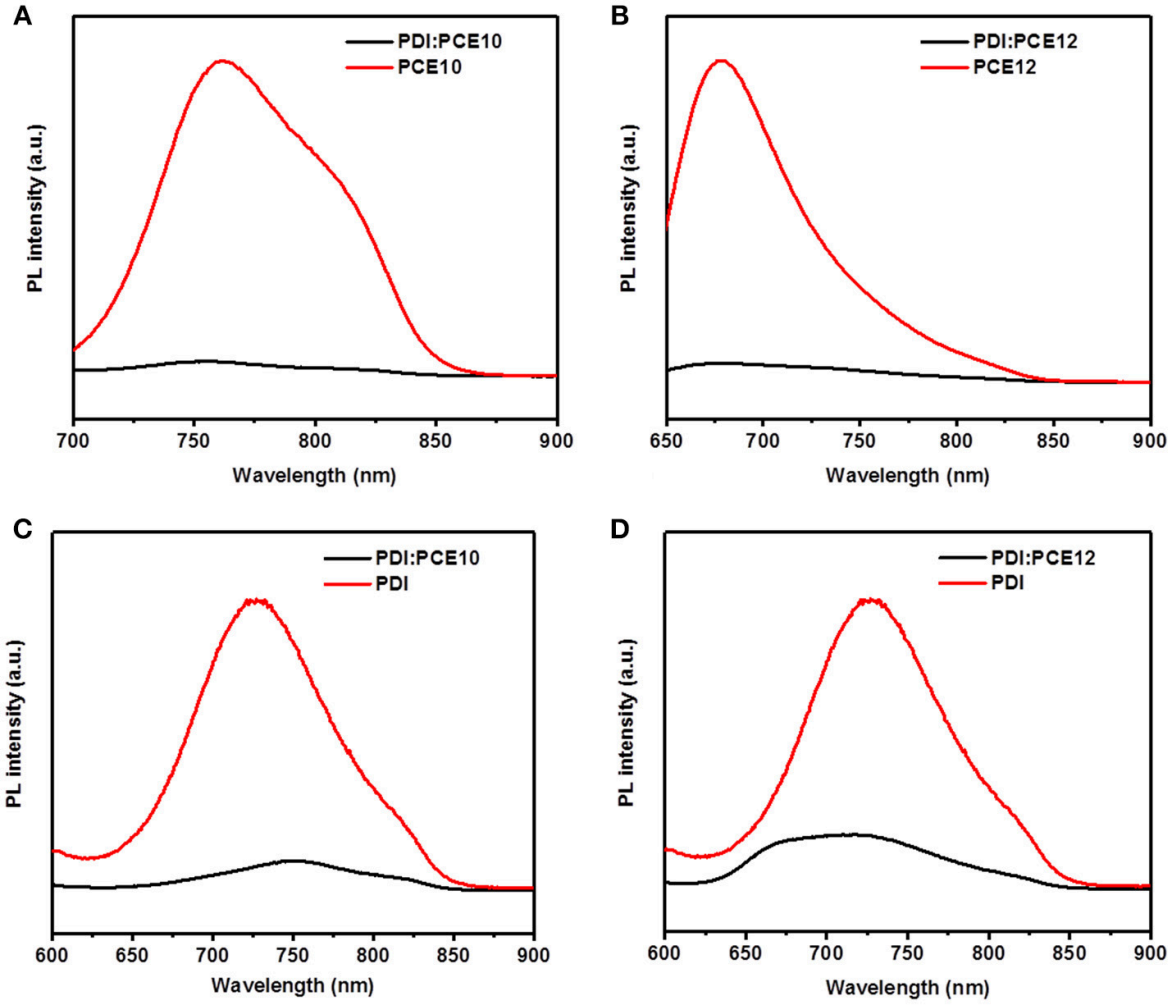
The PL spectra of **(A)** PCE10, PCE10/PDI, **(B)** PCE12, PCE12/PDI, **(C)** PDI, PCE10/PDI, and **(D)** PDI, PCE12/PDI.

The photoluminescence (PL) quenching efficiency is one of the methods to check whether the donor/acceptor combination may work or not. Low quenching efficiency usually relates to large domain size of the donor and acceptor and can translate to severe geminate recombination and poor exciton dissociation efficiency, resulting in bad device performance (Ye et al., 2013; Liu et al., 2016). When the donors and acceptor were photoexcited individually, the PL quenching were shown in Figures 2A–D. As depicted in the Figures 2A,B, PL quenching efficiencies of 95% and 92% were achieved when the donor materials were excited at 680 nm and 620 nm, respectively. The PL quenching efficiencies larger than 90% suggest that the energy loss of geminate recombination in donor materials is secondary. When the acceptor was excited at 550 nm, PL quenching efficiencies of 89% and 77% were obtained as shown in Figures 2C,D. The lower PL quenching efficiencies indicate that the energy loss of geminate recombination in the blends is severe when the acceptor was excited. As presented in Figures 2B,D, PCE12/PDI system behaves lower PL quenching efficiency and more severe geminate recombination when both the donor material and acceptor were excited (Ye et al., 2013).

**Figure 3:**
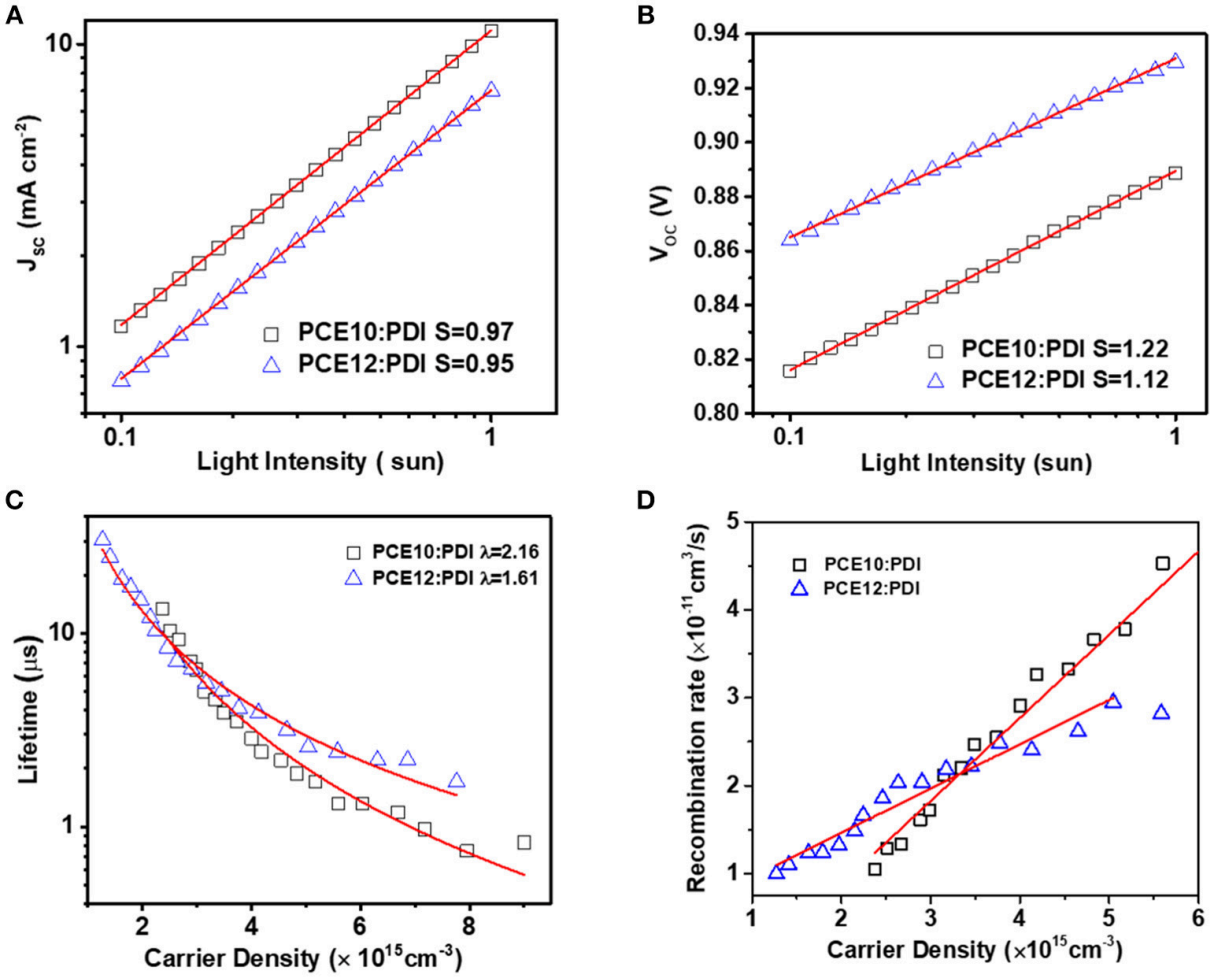
**(A)** J_SC_ vs. light intensity curves, and **(B)** V_OC_ vs. light intensity measurements **(C)** Carrier lifetime as a function of carrier density and **(D)** Recombination rate as a function of charge carrier density. Obtained from TPC and TPV based on PCE10/PDI and PCE12/PDI.

Light intensity dependent J_SC_ and V_OC_ were reported as an easy way to probe the recombination patterns in PSCs. In general, the J_SC_ was plotted against incident light intensity in a log-log scale, following a relationship: J_SC_ ∞ I^α^, whereby 1) α = 1 indicates that all dissociated free carriers are swept out of the device prior to the bimolecular recombination and 2) α < 1 implies a dependence of J_SC_ on bimolecular recombination. As shown in Figure 3A and S3, the α was fitted with a value of 0.95 (±0.01) and 0.97 (±0.01) for PCE12/PDI and PCE10/PDI devices, respectively, suggesting that there was a certain extent of bimolecular recombination in both devices. The comparable α value means that bimolecular recombination was similar in both devices (Cowan et al., 2010; Koster et al., 2011). Next, we fitted the V_OC_/incident light intensity in a natural-log scale to a relationship: V_OC_ ∞ nkT/q ln(I), where k, T, and q are the Boltzmann constant, temperature in Kelvin, and the elementary charge, respectively. The parameter n (usually in the range of 1–2) reflects the presence/absence of carrier traps across the active layers or at interfaces with the electrodes. Any deviations from *n* = 1 (trap-free condition) point to the existence of the effect of trap-assisted recombination. As presented in Figure 3B and S3, the n-values for PCE12/PDI and PCE10/PDI are 1.12 (±0.02) and 1.22 (±0.10), respectively. The n value larger than 1 indicates that trap-assisted recombination is in both devices and the relatively larger n value of PCE10/PDI implies that trap-assisted recombination is more severe in the PCE10/PDI devices (Koster et al., 2005).

To have a deeper understand on the recombination profiles and quantitatively recombination characteristics, we then performed transient photocurrent (TPC) and transient photovoltage (TPV) characteristics (Li et al., 2011). From Figures S4A,B, we noticed that in both systems, the current reached to steady state current within 2 μs without current spike, in both systems, resulting from proper charge generation. By integrating the current after the pulse lights off, the total generated charges can be estimated as discussed later in this part. We further compared the normalized TPC of the two systems at 1 sun condition, and it was found that the current decay in the system of PCE10/PDI was faster than that of the PCE12/PDI system shown in Figure S6, implying that the charge extraction in PCE10/PDI was better, in agreement with the results we made on the mobility measurement. While TPC provides the information on charge generation and extraction at short circuit condition, TPV gives the information on charge carrier lifetime and recombination at open circuit condition. The carrier life time was 1.7 and 0.9 μs for PCE12/PDI and PCE10/PDI systems at V_OC_ condition, respectively. The longer carrier life time of PCE12/PDI indicating reduced recombination loss. As fitted in Figure 3C and S5, the charge carrier lifetime changes with the charge carrier density following the relationship of $\tau =\tau_{0}n^{-\lambda }$, where τ is the carrier lifetime, n is the carrier density, λ is the recombination order. The recombination order λ = 2 implies pure bimolecular recombination in the deceives, and other λ value implies both bimolecular recombination and trap-assisted recombination. We found that λ = 1.61 (±0.04) and λ = 2.16 (±0.10) were obtained from PCE12/PDI and PCE10/PDI systems. Based on the recombination order, the recombination rate then can be calculated as$\;k_{rec}=\frac{1}{\left(1+\lambda \right)n\tau }$ (shown in Figure 3D) (Guo et al., 2013; Li et al., 2016). We can find that the *k*_*rec*_ of PCE12/PDI is lower than that of PCE10/PDI when charge carrier density is larger than 3.3 × 10^15^ cm^−3^. While close to the 1 sun condition, the charge carrier density is far larger than 3.3 × 10^15^ cm^−3^, and then *k*_*rec*_ of PCE12/PDI is far lower than that of PCE10/PDI. Overall, light intensity dependent J_SC_ and TPC indicate the bimolecular recombination was comparable and the charge extraction in PCE10/PDI was better; the light intensity dependent V_oc_ and TPV study suggest that the dominating recombination route in PCE10/PDI device was trap-assisted recombination which leads to a severe recombination rate. One may argue that the recombination rate of PCE12/PDI system was lower than that of the PCE10/PDI thus, it appears to contradict with the better device performance of PCE10/PDI. Here we remind our readers that the electron mobility of PCE12/PDI device is about 5 times slower than that of PCE10/PDI device, so we conclude that the high electron mobility should be a very important factor to give an efficient charge extraction and then the device performance in PCE10/PDI devices.

### Morphology Characterization

**Figure 4:**
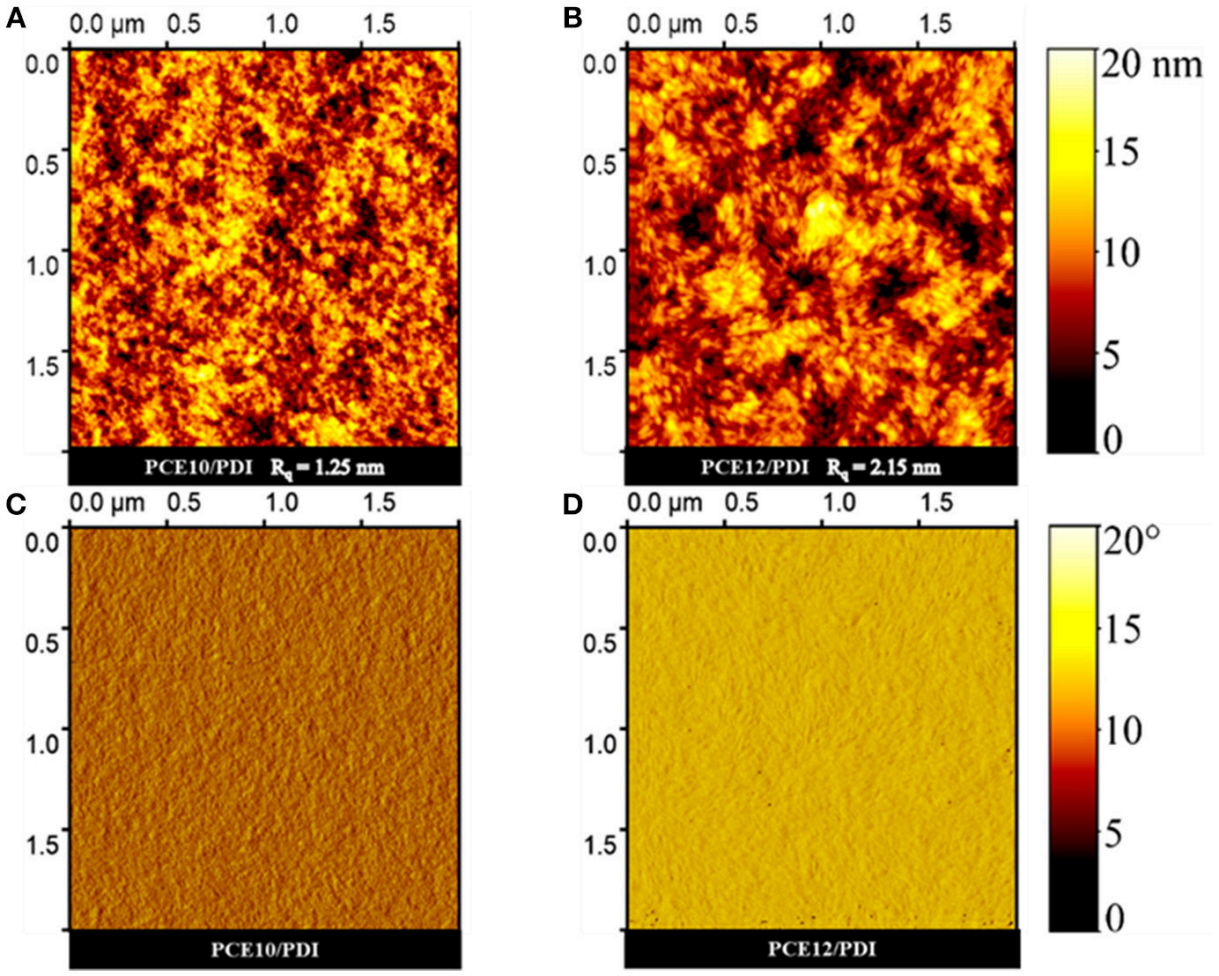
AFM topographical height **(A,B)** and phase images **(C,D)** of PCE10/PDI and PCE12/PDI films.

Finally, we studied the morphological properties of the PCE12/PDI and PCE10/PDI blend films using tapping-mode atomic force microscopy (AFM). The PCE12/PDI film shows a rougher surface (Rq = 1.25 nm) than that of PCE10/PDI (Rq = 1.25 nm, see Figures 4A,B). What's more, larger extend of the phase separation with granular aggregate sizes was observed for PCE12/PDI blend films, as shown in Figures 4C,D and Figure S7. Due to the limited exciton dissociation length (10–20 nm), smaller extent of phase-separation is beneficial for realizing efficient exciton dissociations in the device, suggesting that PCE10/PDI film has a more favourable morphology than that of the PCE12/PDI film. The morphology results well explained the low PL quenching efficiency of PCE12/PDI owing to the strong geminate recombination. The low carrier mobility of PCE12/PDI can also be attributed to the large phase-separation, breaking the continuous pathway for electron transport. Therefore, we can conclude that the PCE12 is tending to form large size aggregations with Bis-PDI-T-EG, which is unfavorable to device performance. While PCE10 presents better compatibility with Bis-PDI-T-EG, favorable phase separation can be expected in the blend. As reported, PCE12 tends to aggregate in films and PCE10 has the tendency of forming amorphous films (Zhao et al., 2016b; Baran et al., 2017, 2018). The differences in the chemical structure of PCE12 and PCE10 should be responsible for the significant difference in thin film morphology, and it is important to check the donor materials' aggregation nature when those were chosen for PSCs with Bis-PDI-T-EG as the acceptor.

## Conclusion

In this work, we systematically compared two polymer/PDI blends on their optical properties, photovoltaic performance, charge carrier transport and recombination, and the thin film morphology. We found that the PCE10/PDI and PCE12/PDI can give a PCE of 5.3 and 3%, respectively, both with FF about ~50–60%. The hole mobilities of both devices are comparable, 3.4 × 10^−4^ and 6.4 × 10^−4^ cm^2^/V s were obtained for PCE12/PDI and PCE10/PDI, respectively. However, the electron mobilities behave 5 times difference, 2.3 × 10^−6^ and 1.2 × 10^−5^ cm^2^/V s were obtained for PCE12/PDI and PCE10/PDI, respectively. The obvious unbalanced charge carrier mobility resulted in low FF of these PSCs. By combining PL quenching efficiency, light intensity dependent J-V measurements, transient photocurrent and transient photovoltage, we noticed that both systems showed a similar extend of bimolecular recombination and PCE10/PDI behaved a severe trap-assisted recombination. Although the recombination rate of PCE10/PDI system was stronger than that of the PCE12/PDI, the high electron mobility and the wide absorption spectrum of PCE10/PDI film result in better device performance in PCE10/PDI devices. The mobility was determined by the distinct aggregation and phase-distribution in the blend. Our findings suggest that it is important to check the aggregation nature of donor materials for Bis-PDI-T-EG based PSCs, and a proper choice is that donor material doesn't tend to aggregate, leading to favorable phase separation.

## Author Contributions

TY, ZK, CZ, and SL proposed the idea of this paper and contributed to analize the experiment results and wring the paper. ZK, TY, and XZ contributed to the fabrication of the solar cells and characterization. SJ, CK, and CT contributed to the synthesis of the Bis-PDI-T-EG acceptor.

### Conflict of Interest Statement

The authors declare that the research was conducted in the absence of any commercial or financial relationships that could be construed as a potential conflict of interest.
