# Supplementary material for: Comparison Study of Wide Bandgap Polymer (PBDB-T) and Narrow Bandgap Polymer (PBDTTT-EFT) as Donor for Perylene Diimide Based Polymer Solar Cells
Source: Front Chem. 2018 Dec 10;6:613. doi: 10.3389/fchem.2018.00613 (PMC6302911; doi:10.3389/fchem.2018.00613)
Supplement: Supplementary file 1 [file Table_1.DOCX]

Supplementary Material

Comparison study of Wide Bandgap Polymer (PBDB-T) and Narrow Bandgap Polymer PBDTTT-EFT) as Donor for Perylene Diimide Based Organic Solar Cells

Tengling Ye^1*^, Shan Jin^1^, Cong Kang^1^, Changhao Tian^1^, Xin Zhang^3^, Chuanlang Zhan^3*^, Shirong Lu^2^, Zhipeng Kan^2*^

^1^MIIT Key Laboratory of Critical Materials Technology for New Energy Conversion and Storage, School of Chemistry and Chemical Engineering, Harbin Institute of Technology, Harbin 150001, P. R. China.

^2^Organic Semiconductor Research Center, Chongqing Institute of Green and Intelligent Technology, Chinese Academy of Sciences, Chongqing 400714, China

^3^Beijing National Laboratory for Molecular Sciences, CAS Key Laboratory of Photochemistry, Institute of Chemistry, Chinese Academy of Sciences, Beijing 100190, P. R. China.

*** Correspondence:**Corresponding Author
[ytl@hit.edu.cn](mailto:ytl@hit.edu.cn), clzhan@iccas.ac.cn, [kanzhipeng@cigit.ac.cn](mailto:kanzhipeng@cigit.ac.cn)

# Experimental details

In this contribution, the donor materials PBDTTT-EFT and PBDB-T were purchased from Solarmer Energy Inc. without further treatment when used. The wide bandgap acceptor Bis-PDI-T-EG were synthesized following previous reports(Zhang et al., 2013). All solutions were prepared in the glovebox using polymers purchased and PDI. Optimized devices were obtained by dissolving the (35mg/ml) polymer / PDI in 1, 2-dichlorobenzene with 2% CN and 2% DIO (volume%) using the polymer/PDI with ratio of 2:3 (wt/wt) for PCE10 and in chlorobenzene with 2% CN and 2% DIO (volume%) using (20mg/ml) polymer/PDI with ratio of 2:3 (wt/wt) for PCE12. The as-prepared solutions were stirred whole night at 115 °C and then decrease to room temperature before being cast.

The solar cells were prepared on glass substrates with tin-doped indium oxide (ITO, 15 Ω sq^−1^) patterned on the surface (device area: 0.1 cm^2^). Substrates were first scrubbed with dilute Extran 300 detergent solution to remove organic residues before immersing in an ultrasonic bath of dilute Extran 300 for 15 min. Samples were rinsed in flowing deionized water for 5 min before being sonicated (Branson 5510) for 15 min each in successive baths of acetone and isopropanol. Next, the samples were dried with pressurized nitrogen before being exposed to a UV−ozone plasma for 20 min. A thin layer (~25nm) of amorphous ZnO (Sun et al., 2011)was spin cast onto the UV-treated samples, dried on the hot plate at 170 °C for 20 minutes, and then transferred into a dry nitrogen glovebox (< 3 ppm O_2_). The active layers were spin-cast at an optimized speed of 2000 rpm in a time period of 45 s, using a programmable spin coater from Specialty Coating Systems (Model G3P-8). After that, the samples of PCE10/PDI were kept for 15 min and then transfer in the lid-covered Petri dish with o-DCB, and the time for solvent annealing were 15min. There is no solvent annealing for PCE12/PDI. The optimized film thickness for PCE10/PDI was 90 nm, and the thickness for PCE12/PDI was 75 nm. Next, the samples were placed in a thermal evaporator for evaporation of 7 nm thickness MoO_3_ evaporated at 0.3 Å s^−1^ and 120 nm of silver electrodes evaporated at 3-4 Å s^−1^; pressure of less than 2x10^-6^ Torr. Following electrode deposition, samples underwent J−V testing.

Solar cell J-V measurements were performed in the glovebox with a Keithley 2400 source meter and an Oriel Sol3A Class AAA solar simulator calibrated to 1 sun, AM1.5 G, with a KG-5 silicon reference cell certified by Newport. The external quantum efficiency (EQE) measurements were performed at zero bias by illuminating the device with monochromatic light supplied from a Xenon arc lamp in combination with a dual-grating monochromator. The reflectance measurements for internal quantum efficiency (IQE) measurements were performed using the same setup for EQE measurements. The number of photons incident on the sample was calculated for each wavelength by using a silicon photodiode calibrated by NIST. All light-intensity dependent of J-V curves, transient photocurrent (TPC), transient photovoltage (TPV) experiments were as reported with pulse light duration was 200 µs(Cowan, Roy et al. 2010, Li, Gao et al. 2011).

Transfer matrix modeling was used to simulate maximum theoretical *J*_SC_ plots as a function of active layer thickness (thickness range: 0-500 nm) for optimized blends of PCE12, and PCE10 with PDI; the model assumes 100% internal quantum efficiency (IQE). The transfer matrix code for these simulations was developed by George F. Burkhard and Eric T. Hoke; code available from: <http://web.stanford.edu/group/mcgehee/transfermatrix/index.html>.

The charge carrier mobility was obtained by fitting the dark current density to the space charge limited current (SCLC) model. The hole and electron only diodes were with device architecture of Glass/ITO/MoO_3_/Active layer/MoO_3_/Ag, and Glass/ITO/ZnO/Phen-NaDPO/Active layer/Phen-NaDPO/Ag, respectively. The build-in voltage was 0 in both cases.

# Supplementary Figures

**Supplementary Figure** 1**.** **(A)** PCE10/PDI simulated current, and **(B)** PCE12/PDI simulated current.

**Supplementary Figure 2.** The charge carrier mobilities of PCE10/PDI (A) and PCE12/PDI (B) blend films determined from single-carrier devices SCLC model.

**Supplementary Figure 3.** **(A)**PCE10/PDI, **(B)**PCE12/PDI.

**Supplementary Figure 4.** **(A)** TPC PCE10/PDI, **(B)** TPC PCE12/PDI.

**Supplementary Figure 5.** **(A)** TPV PCE10/PDI, **(B)** TPV PCE12/PDI.

**Supplementary Figure 6.** Normalized TPC at 1 sun.


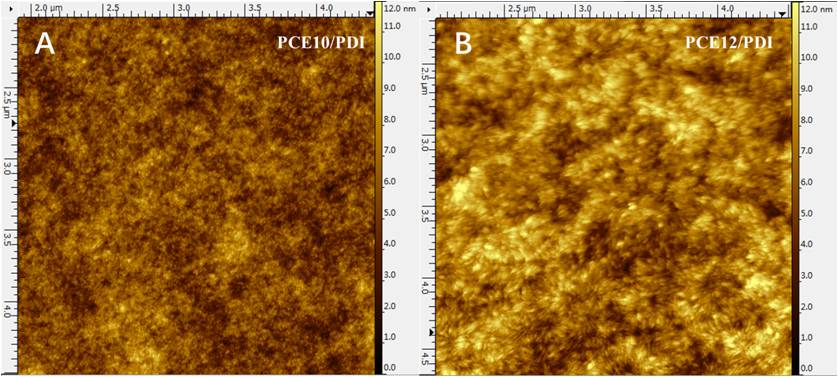


**Supplementary Figure 7.** AFM topographical height images **(A)**, **(B)** in 5μm scale

# References

Cowan, S. R., et al. (2010). "Recombination in polymer-fullerene bulk heterojunction solar cells." *Phys. Rev. B* 82, 245207. doi.org/10.1103/PhysRevB.82.245207.

Li, Z., et al. (2011). "Comparison of the Operation of Polymer/Fullerene, Polymer/Polymer, and Polymer/Nanocrystal Solar Cells: A Transient Photocurrent and Photovoltage Study." *Adv. Funct. Mater.* 21, 1419-1431. doi.org/10.1002/adfm.201002154.

Sun, Y., Seo, J. H., Takacs, C. J., Seifter, J., and Heeger, A. J. (2011). Inverted polymer solar cells integrated with a low-temperature-annealed sol-gel-derived ZnO film as an electron transport layer. *Adv. Mater.* 23, 1679–1683. doi:10.1002/adma.201004301.

Zhang, X., Lu, Z., Ye, L., Zhan, C., Hou, J., Zhang, S., et al. (2013). A potential perylene diimide dimer-based acceptor material for highly efficient solution-processed non-fullerene organic solar cells with 4.03% efficiency. *Adv. Mater.* 25, 5791–5797. doi:10.1002/adma.201300897.
